# Identity by descent and association analysis of dichotomous traits based on large pedigrees

**DOI:** 10.1186/1753-6561-5-S9-S31

**Published:** 2011-11-29

**Authors:** Tian Liu, Anbupalam Thalamuthu

**Affiliations:** 1 Human Genetics Group, Genome Institute of Singapore, 60 Biopolis Street #02-01, Singapore 138672

## Abstract

The goals of our analysis were to map functional loci, which contribute to the case-control status of a trait of interest, using large pedigrees. We used logistic regression fitted with the generalized estimation equation to test associations between a dichotomous phenotype and all genotyped common and rare single-nucleotide polymorphisms. In addition to the association study, we also developed and applied a simple and fast identical-by-descent-based test to identify loci that were shared among affected individuals more often than expected by chance. Among the top significant loci, we assessed the statistical power and the false discovery rate of both methods. We also demonstrated that family-based studies, compared with the standard population-based association studies, have great values and advantages for the discovery of multiple rare causal variants.

## Background

Population-based genome-wide association studies (GWAS) using unrelated individuals are becoming increasingly popular in genetic research. Recent large GWAS have shown that common genetic variants are involved in common diseases, but most of the variants found in this way account for only a small portion of the trait variance. On the other hand, accumulating evidence from candidate-gene-based resequencing suggests that many rare genetic variants contribute to the trait variance of common diseases. Pedigree resources are conventionally believed to be powerful for identifying rare variants and are considered appropriate for the application of linkage strategies. However, linkage analysis often requires nuclear families characterized by multiple informative offspring and is more applicable for quantitative traits [1,2]. Other methods, which exist to appropriately perform association analyses of dichotomous traits with extended pedigrees, are often computationally impractical for large-scale genome-wide association analyses. Hence we are motivated to explore alternative strategies that can be efficiently carried out on a genome-wide scale and can appropriately handle familial relationships in arbitrary-structured pedigrees.

The family data from Genetic Analysis Workshop 17 (GAW17) provide us with a great opportunity to investigate appropriate approaches for the family-based association tests. The approaches we consider in this work include fitting a logistic regression model with adjustments of covariates and a novel approach using identical-by-descent (IBD) measures.

## Methods

We used the family data sets provided by GAW17. This data set had 697 subjects (209 affected and 488 unaffected individuals) from 8 extended families and fully informative markers for 3,205 genes. Assuming that recombination was not allowed within genes, IBD scores were also provided at each gene location (see Almasy et al. [3] for additional details of the GAW17 data). Using the case-control data sets, we explored two different types of methods: association analysis and IBD linkage analysis.

### Association analyses

Throughout this paper, we assume an additive model for the genetic effects. The association analyses were based on the logistic regression model. Preliminary analysis showed that both Age and Smoke are independent risk factors and are significantly associated with the case-control status. Hence the resulting final logistic regression model is:(1)

where *y_i_* is the affected status of individual *i*, case samples are coded 1 and control samples are coded 0, and *g_i_* is the genotype of individual *i* at a given SNP marker. The intercept parameter *μ* is called the base line odds and the *β* parameters represent log odds ratio corresponding to the variables used in the model. Assuming that minor allele *B* is the risk allele, *g_i_* is coded 0, 1, or 2 corresponding to genotypes *AA*, *AB*, or *BB*, respectively.

Because the individuals are no longer independent in the large pedigrees, the joint likelihood function for all individuals has a complicated form. We used the generalized estimation equation (GEE) [4,5] with fixed covariance structure to fit the logistic regression (Eq. (1)). We computed the kinship matrix of the 697 individuals using the kinship program in the R package [6] and used the kinship matrix to specify the covariance matrix in the GEE.

Single-nucleotide polymorphisms (SNPs) with minor allele frequency (MAF) less than 1% were considered rare in our work. In the first stage, we excluded rare SNPs before the association analysis and considered only common causal variants. A rare variant, as part of a group of rare variants in the same gene, is also more likely to contribute to the susceptibility of a disease. However, if we apply logistic regressions directly on the rare variants, the GEE will fail because of the singularity in the correlation matrix. The association methods developed for common variants will have limited efficiency for mapping rare variants unless enormous sample sizes exist. Grouping and collapsing rare variants into meaningful groups (e.g., by functional genes, by pathways) has been shown to be a feasible option to improve efficiency in studying rare variants [7]. Therefore, in the second stage of analysis, we collapsed rare SNPs within the same gene and evaluated their association with the disease trait. We noticed that for 99% of the 3,205 genes, the chance for an individual to carry 4 or more rare alleles in a gene is no larger than 0.0072. Hence we define a combined genotype of a set of grouped rare variants based on the total count of rare alleles as follows:(2)

After assigning a collapsed genotype to grouped rare variants, we evaluate the association between collapsed genotypes and disease outcomes using the logistic model specified in Eq. (1).

### IBD analysis

Phenotypes of relatives are similar because relatives share similar environment and similar genetic variants (genotypes or haplotypes). Genotypes are similar because these relatives share genes that are identical by descent. Intuitively, the disease-associated loci are more likely to be similar in case samples than in control samples. Comparing the distributions of IBD scores between arbitrary pairs of case subjects and pairs of control subjects appears to be a promising approach to identify loci associated with a trait. Based on this general idea, we formulated a new test using a 2 × 3 contingency table that can identify loci shared among affected individuals more often than expected by chance. Our algorithm considers all relationships simultaneously and can be used to test association in pedigrees of arbitrary size.

Suppose that *K* extended families with multiple affected offspring are randomly collected from a natural human population. Consider a SNP marker with two alleles, *A* and *B*, that is genotyped for all individuals in these *K* extended families. For an arbitrary pair of samples *i* and *j* within a family *k*, the IBD score between them at a SNP marker is defined as 0, 0.5, or 1 if 0, 1, or 2 of their shared alleles, respectively, arose from the same allele in an earlier generation. Between any two individuals, there are 14 combinations of their IBD scores and genotypes. We list all 14 states in Table 1 and denote these states by *S*_1_, *S*_2_, …, *S*_14_. Assuming that allele *B* is the disease allele, a pair of individuals shares exactly two copies of inherited *B* alleles only in *S*_1_, exactly one copy in *S*_2_, *S*_4_, and *S*_7_, and one copy with probability 0.5 in *S*_8_.

**Table 1:** IBD configurations for pairs of individuals

State	Description
*S*_1_	*BB*/*BB*; IBD = 1
*S*_2_	*BB*/*BB*; IBD = 0.5
*S*_3_	*BB*/*BB*; IBD = 0
*S*_4_	*BB*/*AB*; IBD = 0.5
*S*_5_	*BB*/*AB*; IBD = 0
*S*_6_	*BB*/*AA*; IBD = 0
*S*_7_	*AB*/*AB*; IBD = 1

*S*_8_	*AB*/*AB*; IBD = 0.5
*S*_9_	*AB*/*AB*; IBD = 0
*S*_10_	*AB*/*AA*; IBD = 0.5
*S*_11_	*AB*/*AA*; IBD = 0
*S*_12_	*AA*/*AA*; IBD = 1
*S*_13_	*AA*/*AA*; IBD = 0.5
*S*_14_	*AA*/*AA*; IBD = 0

Consider a SNP marker with two alleles, *A* and *B*. Between two individuals, if both alleles are identical by descent (IBD), then the IBD score is 1; if one allele is IBD, then the IBD score is 0.5; and if no allele is IBD, the IBD score is 0. For an arbitrary pair of samples within the same family *k*, there are 14 combinations of their IBD scores and genotypes, shown here as *S*_1_ to *S*_14_.

Under the null hypothesis of no association, the frequencies of the 14 states are the same in affected and unaffected samples. Consequently, the pairs of affected and pairs of unaffected individuals have equal frequencies of sharing zero, one, or two copies of *B* alleles IBD. On the other hand, under the alternative hypothesis of association, we expect to observe that pairs of affected individuals share at least one copy of the *B* allele with greater chance. This also means, under the alternative hypothesis, that we expect that affected individuals involved in forming pairs will share at least one copy of the inherited *B* allele with a greater chance than unaffected individuals.

Based on this general idea, we tabulate affected individuals and unaffected individuals by a 2 × 3 contingency table in the following way. The affected individuals can be separated into three groups: Group 1 contains those individuals who form pairs IBD at both alleles; group 2 contains individuals who form pairs IBD at exact one allele and who are not in group 1; and group 3 contains the rest of the individuals. Similarly, unaffected individuals can be assigned to the three groups in the same manner. The cells of Table 2 correspond to the counts of affected and unaffected individuals in the three groups.

**Table 2:** Contingency table

Group		Number of distinct individuals who form pairs IBD at *B*|*B*	Number of distinct individuals who form pairs IBD at *B*|− but not *B*|*B*	Other
Case	Observed	*nBB*^1^	*nB*^1^	*n*^1^
	Expected	*p*_case_(*nBB*^1^ + *nBB*^0^)	*p*_case_(*nB*^1^ + *nB*^0^)	*p*_case_(*n*^1^ + *n*^0^ )
Control	Observed	*nBB*^0^	*nB*^0^	*n*^0^
	Expected	*p*_control_(*nBB^1^* +*nBB^0^*)	*p*_control_(*nB*^1^ + *nB*^0^)	*p*_control_(*n*^1^ + *n*^0^ )

We tabulate affected individuals and unaffected individuals using a 2 × 3 contingency table. Using this table, we formulate our IBD test as a chi-square test. Under the alternative hypothesis, affected samples tend to form more pairs that are IBD at *B*|*B* or *B*|−. In other words, distinct affected individuals have a greater chance of forming pairs IBD at *B*|*B* or *B*|−. Throughout the table, *nBB*^1^/ *nBB*^0^ denotes the number of distinct affected/unaffected individuals who form pairs IBD at *B*|*B*; *nB*^1^/*nB*^0^ denotes the number of distinct affected/unaffected individuals who form pairs IBD at *B*|-, but not *B*|*B*; *n*^1^/*n*^0^ denotes the number of the rest of the affected/unaffected individuals.

**Figure 1:**
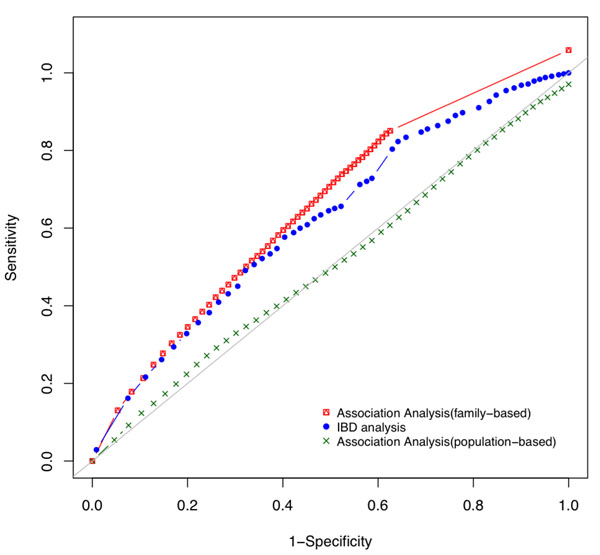
**Receiver operating characteristic curves.** Plot of the true-positive rate against the false-positive rate for the different possible cutpoints of a diagnostic test. These ROC curves are for the two family-based approaches and the population-based association analysis that we performed using GAW17 family-based data sets and population-based data sets. The shape of each curve indicates the quality of the corresponding method. The closer a ROC curve is to the diagonal of the plot, the worse the corresponding method is. A hypothetical perfect method would have a ROC curve that is a constant function with true-positive value 1 and false-positive value 0. The two family-based approaches (the logistic regression using GEE and the IBD analysis) performed significantly better than the standard population-based association study.

Next, we demonstrate this tabulating procedure using a toy. In our example, 13 individuals form two three-generation pedigrees (as illustrated in Figure 1). Individuals 1, 6, 7, 10, 12, and 13 are affected; individuals 2, 3, 4, 5, 8, 9, and 11 are unaffected. Among the affected individuals, individuals 6 and 7 form a pair (6, 7) with two IBD alleles. We assign them to group 1, and the cell count *nBB*^1^ of cell (1,1) is 2. Individuals who form pairs IBD at exact one allele include individuals 1, 6, 7, and 12. Because individuals 6 and 7 have already been assigned to group 1, only individuals 1 and 12 are assigned to group 2. Note that, individuals 10 and 13 form a pair (10, 13) at *S*_8_ (see Table 1). Because they share exactly one copy of IBD allele *B* with probability 0.5, they are assigned to group 2 with probability 0.5. Therefore the cell count *nB*^1^ of cell (1,2) is 2 + 0.5(2) = 3, and the cell count *n*^0^ of cell (1,3) is 0.5(2) = 1. Similarly, among the unaffected individuals, none of them form pairs with two IBD alleles; individuals 2 and 8 form a pair (2, 8), which has one IBD allele; and the rest of the unaffected individuals, individuals 3, 4, 5, 9 and 11, form pairs with zero IBD alleles. Hence the cell counts *nBB*^0^, *nB*^0^, and *n*^0^ of cell (2,1), cell (2,2), and cell (2,3) are 0, 2, and 5, respectively.

Using Table 2, we formulated our IBD test as a chi-square test, and the test statistic is:(3)

Under the null hypothesis *H*_0_, the expected cell frequencies of the affected individuals should be the same as the expected cell frequencies of the unaffected individuals, and the test statistic  has an asymptotic chi-squared distribution with two degrees of freedom.

One great advantage of this IBD-based method is that we can apply it not only to the common variants but also to the rare variants. However, when the MAF (the frequency of the *B* allele) is small, we may observe extremely low cell counts for *nBB*^1^ and *nBB*^0^. Under such circumstances,  no longer follows a chi-square distribution, and it is more appropriate for us to evaluate the significance of the association through permutation testing. We propose to obtain the empirical *p*-values by shuffling the affected statuses of samples within each family. And as a by-product, possible biases caused by different family sizes or family structures will also be corrected. In our toy example, the chi-square test *p*-value (see Table 3 for the observed and expected cell counts) was 0.089, and the resulting empirical *p*-value after 5,000 rounds of permutations was 0.413.

**Table 3:** Contingency table of the toy example

Group		Number of distinct individuals who form pairs IBD at *B*|*B*	Number of distinct individuals who form pairs IBD at *B*|− but not *B*|*B*	Other
Case	Observed	2	3	1
	Expected	(6/13)(2 + 0) = 12/13	(6/13)(3 + 2) = 30/13	(6/13)(1 + 5) = 36/13
Control	Observed	0	2	5
	Expected	(7/13)(2 + 0) = 14/13	(7/13)(3 + 2) = 35/13	(7/13)(1 + 5) = 42/13

## Results

We first compared the performances of two different family-based approaches (association analysis and IBD analysis) in terms of detection power and false discovery rate using the 200 simulated extended family data sets in GAW17. We then carried out standard stratified association analyses with the population-based data sets so that we could further investigate the application and the value of using family-based approaches. In particular, we were keen to find out whether family-based approaches have certain advantages in discovering rare causal variants.

### Family-based association analysis

**Figure 2:**
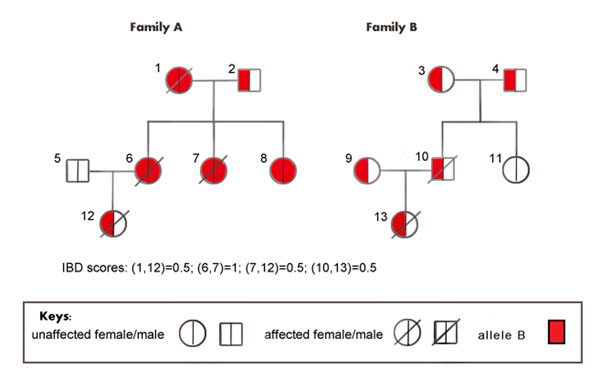
Pedigrees of two hypothetical three-generation extended families

The association analyses were performed by fitting the logistic regression using the GEE on all common SNPs (MAF > 0.01) and combining rare SNPs. Within each gene, we first made corrections for the false discovery rate (FDR) [8]; then we selected the minimum *p*-value to present the gene-level *p*-values. In this way, we were able to make fair comparisons across genes with different sizes. We calculated the correlations of these FDR-corrected [8] gene-level *p*-values for all possible pairs out of 200 rounds of simulations, and the average correlation of these across all 200 rounds of simulations was 0.965, which suggested that the results given by the association analyses were fairly stable. We noticed that the association tests were still inflated with some false positives, as shown in the Q-Q plots (Figure 2b). The estimated inflation factors ranged from 1.02 to 1.36.

We also evaluated false-positive rates. We declared the significance of the gene-level *p*-values with an arbitrary cutoff value *α*. Genes with *p*-values less than 0.05 were considered detected in each simulation. Averaging over 200 replicates, the chance of wrongly declaring noncausal genes (over all 3,205 genes) was 0.096 and 0.033 at an *α* level of 0.05 and 0.01, respectively.

We further evaluated the power of detecting true functional genes at an *α* level of 0.05 and 0.01, respectively. Among all 3,205 genes, the family-based association study successfully identified gene *VEGFA* as the most significant gene with a power as high as 81% at *α* = 0.05. Four other causal genes with the highest discovery rates were *LPL*, *VNN1*, *SHC1*, and *SIRT1*. Compared to the other 31 causal genes, these 5 genes have both strong effects and relatively high frequencies of carrying risk variants.

### IBD analysis

**Figure 3:**
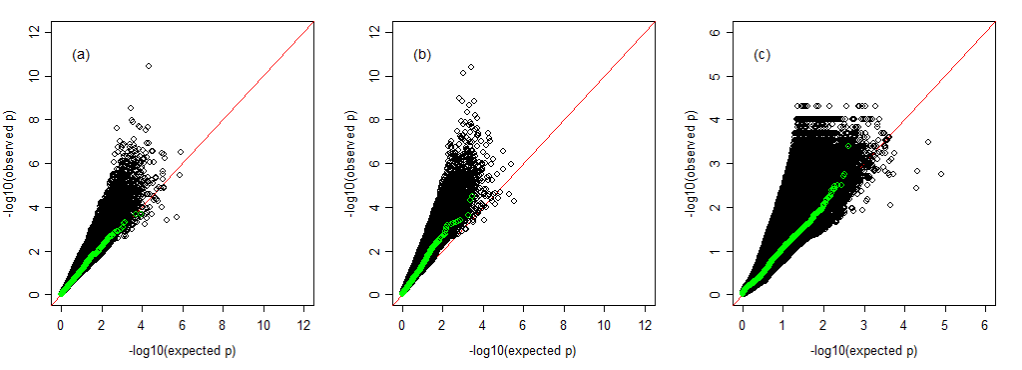
**Q-Q plots of three analyses using population data sets and family data sets**. The panels display Q-Q plots of FDR-corrected gene-level *p*-values from all 200 replicates. Green dots are based on replicate 5. (a) Population-based case-control association studies, using the stratified analysis. (b) Family-based association analyses obtained by fitting logistic regression using the GEE. (c) Family-based IBD analyses, in which raw *p*-values of all rare and common variants are empirical *p*-values. These empirical *p*-values are based on 10,000 rounds of permutations.

The proposed IBD test was performed on all common and rare SNPs. Raw *p*-values of all SNPs were the empirical *p*-values based on 10,000 permutations. Within each gene, the FDR-corrected minimum *p*-values were used to present the gene-level significance. As shown in Figure 3, the sensitivity of the IBD analysis approach was comparable to the association analysis approach. However, the results from the proposed IBD test were not as stable as the results from the association analyses; the average correlation of the FDR-corrected gene-level *p*-values across all 200 rounds of simulations was 0.461. As shown in the Q-Q plot in Figure 3c, the IBD analyses were inflated with a certain amount of false positives in most of the replicates, and in other replicates our IBD tests were underpowered. The estimated inflation factors of the IBD tests ranged from 0.86 to 1.41, and the actual false-positive rates at *α* = 0.05 and *α* = 0.01 were 0.112 and 0.039, respectively. The five causal genes with the highest discovery rates were *VEGFC*, *SHC1*, *VEGFA*, *HIF3A*, and *SIRT1*. At a significance level of 0.05, our IBD test successfully picked up genes *VEGFC* and *SHC1* with a power of 82% and 67%, respectively, which ranked them first and fifteenth out of all 3,205 genotyped genes.

### Comparing family-based approaches with the population-based association analysis

As a comparison to the family-based analyses, we also performed a standard stratified analysis on the 200 simulated population case-control data sets. We assessed the power of population-based studies for all causal genes based on the outputs from the PLINK computer program. *FLT1*, which has three common variants and large genetic effects, is the only gene with a detection power greater than 50%. For most other causal genes with rare variants, family-based studies had better power of detection than population-based studies. The Q-Q plot in Figure 3a shows that the standard stratified analyses were also inflated with some false positives. The estimated inflation factors ranged from 1.02 to 1.37, and the actual false-positive rates at *α* = 0.05 and *α* = 0.01 were 0.089 and 0.029, respectively. Although all three types of analyses have comparable false-positive rates, the receiver operating characteristic (ROC) curves (Figure 1) show that the two family-based analyses clearly performed significantly better than the standard population-based association study in terms of having higher sensitivity.

## Conclusions

In this work, our analyses provide new insights into the genetic studies of family data with large extended pedigrees for dichotomous traits. We tested the utility of two types of family-based approaches: a logistic regression fitted using GEE and our proposed IBD test. The logistic regression with GEE was used to test the associations in large pedigrees while simultaneously controlling for environmental covariates. To properly handle the rare variants, we provide an operable and straightforward scheme to collapse the rare variants within a gene. This simple collapsing strategy was shown to be useful. As an example, we had a reasonable power for identifying causal gene *SIRT1*, which is enriched with rare causal variants. We also showed that the linkage of disease alleles can be tested based on IBD scores in extended pedigrees. By incorporating information from not only parents and siblings but also other ancestors, we developed a chi-square test based on a 2 × 3 contingency table. Our IBD test provides an attractive alternative to the conventional tests because it is computationally fast and does not require one to specify an inheritance model explicitly.

We compared the performance of family-based studies using these two approaches with the performance of the population-based studies using the standard stratified analysis. Population-based studies seemed to have better power for detecting common variants, and the family-based studies seemed to have better power for detecting rare variants. If a risk allele is present in early founders and the effects of risk alleles are relatively large, the IBD analyses clearly outperformed the family-based association analyses. We noticed that, even for rare variants that have extremely low frequencies or that are found in only a few families, the IBD analysis has a good chance of picking up them. For example, in this work, the power of detecting risk genes *VEGFC* and *HIF3A* using the IBD tests was significantly higher than the power obtained using the logistic regressions. In other instances, family-based association analyses had better power to detect genes that have relatively high frequencies of risk alleles and relatively large genetic effects. In the worst scenario, if the risk allele was extremely rare, not present in early founders, and of small genetic effect, both methods failed. Because the two family-based approaches have their own advantages for dealing with rare variants, these two approaches can potentially compensate each other. Combining these two types of analysis may be a more powerful solution in the search for causal variants.

Although hunting for rare causal variants using family data sets seems promising, many practical issues need to be addressed before the effectiveness of family-based analyses can be fully recognized. For example, the IBD test can produce unstable results, and it is not very straightforward to obtain the gene-level IBD scores in the first place. Also, we noticed that all analyses were inflated with some false positives. We suspect that the inflated false positives may be caused by those nonfunctional variants whose genotypes are highly correlated with the functional variants. Luedtke et al. [9] offers a detailed discussion of these so-called spurious associated genes. Other possible sources of inflated false positives in practical studies include insufficient correction of population stratification, inappropriate handling of rare variants, and the effects of linkage disequilibrium structures. We will further investigate the influence of these possible sources in our future studies.

## Competing interests

The authors declare that there are no competing interests.

## Authors’ contributions

TL and TA conceived the project. TL performed the statistical analysis. Both the authors read and approved the final manuscript.
